# VEB-1 extended-spectrum β-lactamase-producing multidrug-resistant *Proteus mirabilis* sepsis outbreak in a neonatal intensive care unit in India: clinical and diagnostic implications

**DOI:** 10.1099/jmmcr.0.005056

**Published:** 2016-08-30

**Authors:** Sarika Jain, Rajni Gaind, Charu Kothari, Rachna Sehgal, A. Shamweel, S. S. Thukral, Harish K. Chellani

**Affiliations:** ^1^​ Department of Microbiology, VMMC and Safdarjang Hospital, New Delhi 110029, India; ^2^​ Department of Pediatrics, VMMC and Safdarjang Hospital, New Delhi 110029, India; ^3^​ Department of Microbiology, Vallabhbhai Patel Chest Institute, New Delhi, India

**Keywords:** Infection control, Neonatal sepsis, Outbreak, *Proteus mirabilis*, VEB-1 ESBL

## Abstract

**Introduction:**

Extended-spectrum β-lactamase (ESBL)-producing Enterobacteriaceae, multidrug-resistant (MDR) pathogens, are increasingly implicated in nosocomial outbreaksworldwide, particularly in neonatal intensive care units (NICUs). *Proteus mirabilis* is an uncommon nosocomial pathogen causing sepsis in neonates.

**Case Presentation:**

We report an outbreak of ESBL-positive MDR *P. mirabilis* sepsis involving five babies within 10 days in a NICU, which was promptly detected and managed. The aim of this study was to characterize the molecular mechanism of resistance to third-generation cephalosporins (3GCs) in the bacteria. Surveillance cultures were collected from health-care personnel (hand swabs, urine) and the surrounding patient-care environment. Ribotyping was performed to determine the clonality of the strain. Thirteen *P. mirabilis* were recovered from the blood cultures of the five babies and surveillance cultures. Twelve isolates were positive for the VEB-1 ESBL type, and were susceptible only to ciprofloxacin and carbapenems. There was an unusual phenotypic synergy observed between the 3GCs and imipenem/cefoxitin. The source of infection was traced to a contaminated multidose vial. The outbreak was associated with a high mortality (80 %). A change of empirical antibiotic policy to ciprofloxacin, with strict infection control measures, brought the outbreak to an abrupt end.

**Conclusion:**

This is believed to be the first report of a nosocomial outbreak of VEB-1 ESBL-producing *P. mirabilis* sepsis in neonates from India. The present report of infection due to VEB-1-producing *P. mirabilis*, an uncommon pathogen for an epidemic in a neonatal unit, highlights the growing significance of such Gram-negative bacteria as a cause of infections in newborns. Epidemic spread in a neonatal unit of an ESBL-producing *Proteus* species, which also had an intrinsically reduced susceptibility to imipenem, and resistance to colistin and tigecycline, can be a threatening situation and can result in high neonatal mortality unless recognized and controlled in a timely manner.

## Introduction

Neonatal sepsis is a significant cause of morbidity and mortality among newborns in developing countries requiring prompt diagnosis and treatment. Extended-spectrum β-lactamase (ESBL)-producing Enterobacteriaceae are among the multidrug-resistant (MDR) pathogens in hospitals, and outbreaks of ESBL-positive *Escherichia coli* and* Klebsiella pneumoniae* are reported worldwide in neonatal intensive care units (NICUs) (Tschudin-Sutter *et al.*, 2010; Ayan *et al*, 2003; Gastmeier *et al.*, 2007; Johnson, 2011; Velasco *et al.*, 2009). The most prevalent ESBLs among Enterobacteriaceae are TEM-type enzymes (Bradford, 2001); others are SHV, CTX-M and PER-1 (Roy *et al.*, 2013; Livermore *et al.*, 2007), with recent reports of VEB-1 type (Naas *et al.*, 2000; Poirel *et al.*, 1999). Molecular characterization of EBSL types among *E. coli* and *K. pneumoniae* from neonatal sepsis cases at Safdarjung Hospital has shown CTX-M-15 as the most prevalent type, with other types being TEM-1, SHV-1, SHV-28, SHV-11 and SHV-12 (Roy *et al.*, 2013). VEB-1 had not been reported from India.

*Proteus mirabilis* is an uncommon nosocomial pathogen and bacteraemia due to *P. mirabilis* is relatively rare (Diekema *et al.*, 2000; Fluit *et al.*, 2000). *Proteus* species accounted for only 6.6 % of Gram-negative neonatal sepsis cases in a previous study from Safdrjung Hospital (all ESBL producers) (Sehgal *et al.*, 2007), and 3.5 % in another report from North India (Bhattacharjee *et al.*, 2008). There are no reports of neonatal outbreaks due to *Proteus* species from India. ESBL production among *P. mirabilis* is reported worldwide, with a relatively high prevalence of 40 % in some areas (Endimiani *et al.*, 2005), which is a matter of serious concern as resistance to third-generation cephalosporins (3GCs), along with higher MICs (1–4 µg ml^−1^) for imipenem, and intrinsic resistance to reserve antimicrobials like colistin and tigecycline, can result in difficult-to-treat *Proteus* infections (Park & Parker, 1986).

In the present report, we describe an outbreak of VEB-1 ESBL-producing *P. mirabilis* septicaemia in a NICU, which was promptly detected and managed. The aim of this study was to characterize the molecular mechanism underlying the high-level β-lactam agent resistance, determine the clonality, source and routes of transmission of the *P. mirabilis* strain in the neonatal unit and to review the characteristics and outcomes of the neonatal septicaemia cases.

## Case report

**Fig. 1.**
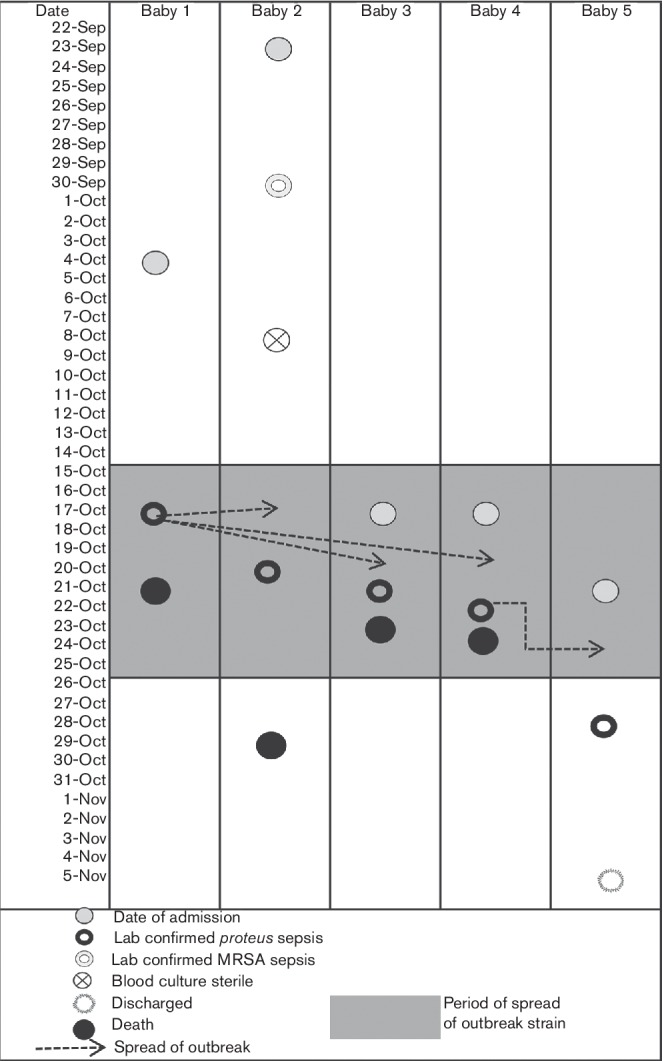
Diagram depicting the timeline of the outbreak and transmission routes between affected babies.

Overall, five cases of ESBL-positive *P. mirabilis* septicaemia were reported over a period of 10 days (Fig. 1). All the five babies had late-onset sepsis (LOS). A review of laboratory data showed there were no cases of *Proteus* septicaemia reported from this neonatal unit during the 4 years preceding this outbreak. When the third strain of *P. mirabilis* was isolated from the same unit, clinicians were alerted regarding a possible outbreak. The clinical details of these babies and associated risk factors for LOS are shown in Table 1.

**Table 1.** Clinical presentation of babies infected with VEB-1 ESBL-producing *P. mirabilis* during the outbreak in a neonatal unit

Isolate no.	Age at onset of sepsis /sex	Risk factor	Antibiotics used prior to sepsis with duration(days )	Antimicrobial resistance	Outcome
1	14 days/M	Prematurity (36 week), LBW (1600 g), PROM	A 5, Ak 5	A, CTX, CRO, CAZ, Ak, G, Nt, TMP-SMX	Died
2	28 days /M	LBW (1200 g), PROM, respiratory distress	A 7, Ak 7	A, CTX, CRO, CAZ, Ak, G, Nt, TMP-SMX	Died
3	5 days /F	ELBW (940 g), prematurity (30 week)	CTX 1, Ak 1	A, CTX, CRO, CAZ, Ak, G, Nt, TMP-SMX	Died
4	6 days /M	ELBW (1100 g), prematurity (30 week)	CTX 2, Ak 2	A, CTX, CRO, CAZ, Ak, G, Nt, TMP-SMX	Died
5	8 days /F	LBW (1300 g), prematurity (34 week), PROM, respiratory distress, DVET	A 7, Ak & CTX 3, Ak 3	A, CTX, CRO, CAZ, Ak, G, Nt, TMP-SMX	Survived

F, Female; M, Male; , DVET, double volume exchange transfusion; ELBW, extremely low birth weight; LBW, low birth weight; PROM, prolonged rupture of membranes; A, ampicillin; Ak, amikacin; CAZ, ceftazidime; CRO, ceftriaxone; CTX, cefotaxime; G, gentamicin; Nt, netilmycin; TMP-SMX, trimethoprim/sulphamethoxazole.

The neonatal unit of Safdarjang Hospital, a tertiary care hospital with a delivery rate of 15 000–18 000 per year and about 20 % of the newborns delivered requiring admission, has two nurseries equipped with 20 beds for intermediate care and 6 beds for intensive care of preterm neonates or infants with serious medical or surgical pathologies requiring intensive care. This NICU is a referral centre for high-risk neonates. Isolation facilities are available. The nurse to patient ratio is 1 : 4–6 in intermediate care (level II) and 1 : 2 in the intensive care unit (level III).

## Investigations

### Laboratory identification and antibiotic susceptibility of bacterial strains

Blood cultures from neonates were drawn and incubated in trypticase soya broth containing sodium poly sulphonate (SPS) at 37 °C and inoculated onto 5 % sheep blood agar (SBA) and MacConkey agar at 24 h, 48 h and 5 days. Informed consent was obtained from the parents of the neonates. Surveillance specimens for culture were collected, which included 50 hand swabs (with sterile cotton swabs) and urine samples of health-care providers (HCPs) in the NICU, 30 environmental samples from various parenteral solutions (such as heparin, dextrose, isolyte-P, gentamicin and povidone iodine), water samples from humidifiers, water used for oxygen and ventilation tubing (all collected in sterile vials), and swabs from resuscitation equipment, suction apparatus, sinks, surfaces, floors and bedding. Rectal swabs or faecal specimens of HCPs could not be obtained. All the samples were incubated overnight in brain heart infusion broth (BHIB), subsequently subcultured onto SBA and MacConkey agar and incubated in air at 37 °C. The organisms were identified by standard methods and confirmed by a Vitek 2 system (bioMérieux). The isolates were subjected to antibiotic susceptibility testing, PCR for the β-lactamase gene, ribotyping and conjugal transfer.

Antimicrobial susceptibility was performed using Kirby Bauer’s disc diffusion method and interpreted as per Clinical and Laboratory Standards Institute criteria (CLSI, 2012). The MIC values (µg ml^−1^) for selected antimicrobials were determined using Etests (AB Biodisk) and interpreted according to the manufacturer's instructions. *E. coli* ATCC 25922 and ESBL-positive *K. pneumoniae* ATCC 700603 were used as controls. The ESBL phenotype was detected by combined disc method (cefotaxime/clavulanic acid and ceftazidime/clavulanic acid) (CLSI, 2012) and was again confirmed by ESBL Etest using both ceftazidime and cefotaxime with and without clavulanic acid.

PCR amplification for *bla*_SHV_, *bla*_TEM_, *bla*_OXA-1_ and *bla*_CTX-M_ was performed for isolates found to be positive in the ESBL confirmatory test as described previously (Roy *et al.*, 2013). PCR for the VEB-encoding gene was performed by using primers 5′-CGA CTT CCA TTT CCC GAT G-3′ and 5′-GGA CTC TGC AAC AAA TAC GC-3′. The cycling conditions were as follows: an initial denaturation at 94 °C for 5 min; followed by 30 cycles consisting of 94 °C for 30 s, 58 °C for 30 s and 72 °C for 45 s; and a final extension step at 72 °C for 10 min. PCR products were sequenced as per the manufacturer’s guidelines in a 3130xl genetic analyser (Applied Biosystems). Furthermore, the nucleotide and deduced amino acid sequences were analysed and compared with sequences available in GenBank at the National Center for Biotechnology Information (NCBI) web site (http://www.ncbi.nlm.nih.gov/genbank).

Ribotyping was performed on *P. mirabilis* isolates from septicaemic neonates, the environment and HCPs, employing DIG-labelled cDNA probe synthesized by using 16S+23S rRNA of *E. coli.* The ribopatterns were analysed using Diversity Database software (Pdi) incorporated in a gel documentation system. Dendograms were reconstructed using GelCompar and the UPGMA clustering method, to find the percentage of similarity between ribopatterns of isolates of *P. mirabilis*.

Conjugal transfer of a 3GC resistance marker to a sodium azide-resistant *E. coli* J53 recipient was attempted by a broth mating assay as described previously (Roy *et al.*, 2013).

*P. mirabilis* (*n*=9) was isolated from paired blood cultures of four babies and a single blood culture of one baby. Antimicrobial susceptibility testing of all *P. mirabilis* isolates from the five babies involved in the outbreak indicated resistance to ampicillin, extended-spectrum cephalosporins (ceftazidime and cefotaxime, MIC >32 µg ml^−1^), cefepime (MIC 24 µg ml^−1^), gentamicin (MIC>32 µg ml^−1^), amikacin (MIC >32 µg ml^−1^) and netilmicin, but susceptibility to ciprofloxacin (MIC 0.125 µg ml^−1^), ertapenem (MIC 0.064 µg ml^−1^), imipenem (MIC 1.00 µg ml^−1^), meropenem (MIC 0.032 µg ml^−1^), piperacillin/tazobactam and cefoperazone/sulbactum (Table 1). All isolates were found to be ESBL producers.

Surveillance cultures yielded *P. mirabilis* from four specimens, one isolate each from a multidose in-use dextrose solution used for diluting antibiotics, an intravenous catheter exit site of the fifth baby, a hand swab of an attending staff nurse and a urine culture of a resident doctor. Sealed parenteral dextrose solution of the same batch was sterile. The antibiograms and MICs of all the isolates from surveillance culture except for the isolate from urine (*n*=3) were identical to the blood culture isolates from the five babies (*n*=9) and all were ESBL positive. The isolates were sensitive to cefoxitin (MIC 2.0 µg ml^−1^) indicating the absence of AmpC β-lactamase. The urine culture isolate differed from other isolates in being susceptible to all antibiotics tested and negative for ESBL enzyme.

**Fig. 2.**
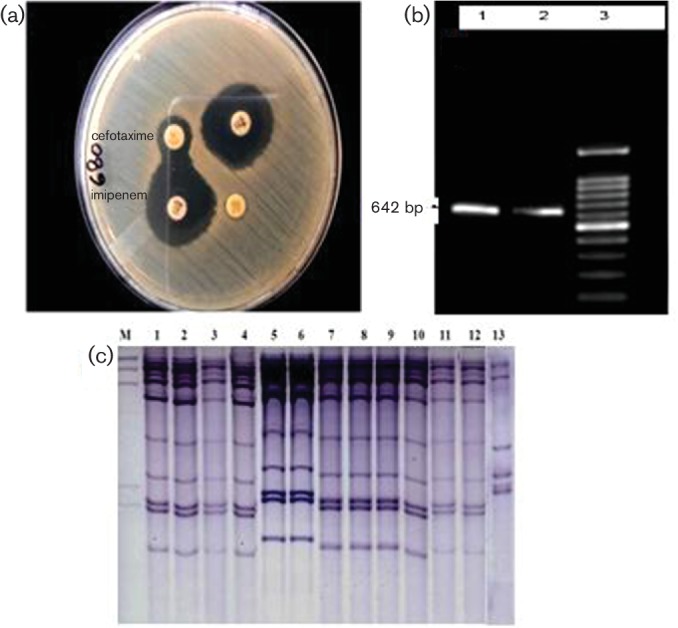
(a) Synergy between imipenem and cefotaxime discs. (b) PCR amplicon of 642 bp in size identified as the VEB-1-encoding gene on sequencing. (c) *Eco*R1 ribotype patterns of the 13 isolates of *P. mirabilis*. Lanes: M, DIG-labelled lambda DNA cleaved with *Hind*III; 1–12, the 12 isolates showing similar ribopatterns; 13, the isolate from urine that had a distinct pattern.

All 12 ESBL-positive *P. mirabilis* isolates exhibited an unusual synergy between 3GCs (cefotaxime and ceftazidime) and imipenem/cefoxitin (Fig. 2a). PCR was positive for *bla_TEM_* (858 bp amplification product) and *bla*_VEB _(642 bp amplicon; Fig. 2b) in all 12 isolates, which were further confirmed by sequencing as TEM-1 and VEB-1 β-lactamases. Experiments involving both conjugative transfer of cefotaxime/ceftazidime resistance and plasmid isolation failed, suggesting that the gene was not located on a conjugative plasmid. Ribotyping demonstrated an identical ribopattern of all nine clinical and three surveillance isolates, indicating that these isolates belonged to the same clone (Fig. 2c).

## Treatment

Empirical antibiotic therapy with cefotaxime for LOS resulted in a poor outcome in the babies. Of the five cases diagnosed, four newborns had a fulminant course of disease and succumbed to the infection. The fifth baby developed ESBL-positive *Proteus* septicaemia 1 week after the fourth case, by then, in view of the outbreak alert, empirical antibiotic therapy for LOS had been modified to ciprofloxacin from cefotaxime and amikacin.

Compliance to infection control practices such as hand-washing, asepsis in intravenous catheter insertion, preparation and administration of intravenous medications, and procedures for routine care of the baby (cord care, bathing, feeding) were reviewed. Evaluation of health-care practices during a 10 day silent observation after the onset of the outbreak revealed that nursing and medical personnel consistently washed their hands before entering the nursery. However, hand-washing was at times not stringently practiced in between patient contact. Aseptic techniques were also suboptimal, especially during night shifts, while performing invasive procedures and reconstitution of antibiotics. In service education sessions with the staff were held reinforcing proper hand hygiene, aseptic precautions while placing or handling intravenous lines, barrier nursing, environmental cleaning, routine use of gloves and gowns for any patient contact, proper cleaning, and sterilization and disinfection of equipment and medical devices. New babies admitted to the unit were nursed in a separate room. All patient care equipment was thoroughly cleaned and disinfected.

The fifth baby responded well to ciprofloxacin therapy and was discharged after 10 days. As a result of strict standard and contact precautions re-instituted in the NICU, no further case of *P. mirabilis* sepsis occurred thereafter.

## Discussion

In the neonatal outbreak reported here, all the five babies had low birth weight and four babies were preterm, predisposing them to the infection. The outbreak was caused by a MDR* P. mirabilis* strain with a high mortality rate (80 %). The only other outbreak of infection due to *P. mirabilis* in a nursery was reported in 1962 involving five newborns over 4 months (Becker, 1962). Furthermore, the VEB-1 ESBL type identified for our outbreak strain has not been described among *Proteus* species from neonates before. VEB, like other ESBL types, is susceptible to clavulanic acid inhibition, but remains under-reported as molecular characterization is not performed routinely in diagnostic laboratories to screen for this gene. However, an unusual synergy observed between imipenem/cefoxitin and 3GCs in all the VEB-producing isolates can be used as a screening tool for recognition of this rare ESBL type. Interestingly, unlike most ESBLs, which are plasmid borne, previous reports demonstrated that the VEB-1-encoding gene is located in chromosomally encoded class 1 integrons (Naas *et al.*, 2000). Experiments for the presence of *bla*_VEB_ on the chromosome were not performed in the present study; however, the resistance to 3GCs conferred by VEB-1 was not transferrable by conjugation tests. Association of a VEB-encoding gene with *arr2* or *aadB* gene cassettes on the integron confers resistance to rifampicin and aminoglycosides (Naas *et al.*, 2001). These genes were not screened by PCR in our study; however, our isolates remained resistant to the aminoglycosides tested.

VEB-1 was first described in an *E. coli* isolate from Vietnam in 1998 (Naas *et al.*, 2000), and since then has been reported from the Far East, South-East Asia, Europe and America among diverse Enterobacteriaceae, *Pseudomonas aeruginosa* and *Acinetobacter baumannii* underlining its interspecies spread (Naas *et al.*, 2000, 2006a, 2006b; Girlich *et al.*, 2002; Pasterán *et al.*, 2006; Woodford *et al.*, 2008; Poirel *et al.*, 2001). Nosocomial outbreaks due to a strain with a VEB-encoding gene are rare and have been predominantly reported in ICUs and hospitalized adult patients (Table 2).

**Table 2.** Review of outbreaks due to VEB ESBL-producing Gram-negative organisms worldwide

Organism	Location	Age	No. of patients	Diagnosis	Country	Reference
Enterobacteriaceae (*Escherichia coli*, **Enterobacter* cloacae,* *Enterobacter sakazakii, Klebsiella pneumonia*e)*	ICU, hospitalized	All age groups including neonates	16	UTI, pustular and biliary infection	Thailand	Girlich *et al.* (2001)
*Acinetobacter* *baumannii*	ICU	Adults	12	Sepsis, pneumonia		Poirel *et al.* (2003)
*Proteus mirabilis*	Hospitalized	Adults	12	Pneumonia, UTI		Kim *et al.* (2004)
*Enterobacter cloacae*†	Hospitalized	Adults	12	Clinical infections	China	Jiang *et al.* (2005)
*Acinetobacter baumannii*	ICU	Adults	275	Clinical infections	France	Naas *et al.* (2006b)
*Acinetobacter baumannii*	Hospitalized	Adults	6	Clinical infections	Belgium	Naas *et al.*, 2006a)
Acinetobacter baumannii*	Hospitalized	Not specified	10	Clinical infections	Argentina	Pasterán *et al.* (2006)
*Pseudomonas aeruginosa*‡	ICU	Not reported	15	Not reported	UK	Woodford *et al.* (2008)
*Providencia* sp.*	Hospitalized	Adults	20	Clinical infections	Tunisia	Lahlaoui *et al.* (2011)
*Pseudomonas* aeruginosa*	Not reported	Not reported	20	Not reported	Riyadh, Saudi Arabia	Al-Agamy *et al.* (2012)

ICU, Intensive care unit; UTI, urinary tract infection.

*Outbreak/epidemic could not be ruled out.

†VEB-1-like ESBL (VEB-3) along with CTX-M.

‡Serotype 015 co-producing *bla*_VEB-1a_ and VIM-10.

Contact is the most frequent mode of nosocomial transmission and studies show that horizontal transmission of Enterobacteriaceae usually occurs through the hands of HCWs (Waters *et al.*, 2004). Maintaining the recommended patient staff ratio (one nurse for every one baby for intensive care and every four babies for intermediate care) is critical in NICUs where neonates are often premature, with vascular lines *in situ*, and are mechanically ventilated, to allow time to wash hands in between patient contact. Other sources implicated in nosocomial outbreaks in nurseries include failures in sterilization/disinfection of multi-use instruments, pooling or multiple use of single-use vials, inappropriate storage of multiple-dose vials, lack of aseptic techniques during invasive procedures, and inadequate hand hygiene and glove use (Zaidi *et al.*, 2005). Among the surveillance culture samples screened, the outbreak strain of *Proteus* was isolated from the intravenous exit site of the fifth baby in the series, a multidose dextrose parenteral solution used for diluting antibiotics and the hands of an attending staff nurse. The nursing personnel may have inadvertently introduced the organism into the bloodstream of the babies while handling intravenous catheters or through infusion of a contaminated diluent of dextrose solution, suggesting a horizontal transfer of the outbreak strain due to a lapse in hand washing and aseptic techniques. To prevent further spread of the strain, improved hand-hygiene compliance, barrier nursing, cohorting of infected babies and restricted use of multi-use medications/equipment were reinforced. These interventions have been shown to be the most important, yet the least expensive, infection control activities in neonatal units (Zaidi *et al.*, 2005). All the babies were assigned individual medication vials and instruments, and a policy of single-use vials and closed intravenous systems for each baby was implemented henceforth.

As the outbreak strain was ESBL positive, another factor that brought the outbreak under control was the change in empirical antibiotic therapy for LOS from 3GCs to ciprofloxacin. Besides ciprofloxacin, the outbreak strain was also susceptible to β-lactam/β-lactamase inhibitor combinations and carbapenems. *P. mirabilis*, *P. vulgaris*and *Morganella morganii* often have MICs just at the carbapenem susceptible breakpoint of 1 mg ml^−1^ (Park & Parker, 1986). In the current study, the MIC of imipenem for *P. mirabilis* strains was also 1 µg ml^−1^. Although the isolates were meropenem susceptible (MIC 0.032 µg ml^−1^), these drugs, being expensive, remain unavailable in resource-limited countries. β-Lactam/β-lactamase inhibitor combinations are subject to increased MICs as the inoculum rises in infections with high bacterial load, such as sepsis, and result in therapeutic failures (Paterson & Bonomo, 2005). We have previously used ciprofloxacin in septicaemic neonates with success, which is a reserve drug for multiresistant pathogens in NICU at Safdarjung Hospital (Sehgal *et al.*, 2007).

### Conclusion

The present report of an outbreak due to VEB-1-producing *P. mirabilis*, an uncommon pathogen for an epidemic in a neonatal unit, highlights the growing significance of such Gram-negative bacteria as a cause of newborn infections and the rapid dissemination of infection in the neonatal unit due to lapses in basic infection-control practices. The number of cases involved in the outbreak was limited, as a result of prompt detection and control of the outbreak by a multipronged approach involving strict infection-control practices and an alteration of the empirical treatment. Sepsis due to ESBL-producing *Proteus* species poses a serious therapeutic threat with limited treatment options and is associated with high mortality rates. Early identification is important to reduce neonatal mortality, and prevent further spread and establishment of endemicity of such strains in the hospital. VEB ESBL can be easily identified in clinical microbiology laboratories by a phenotypic test to detect the unusual synergy between imipenem and 3GCs. Ciprofloxacin can be used as a reserve drug in such infections.

## Acknowledgements

We thank Bianca Paglietti and Professor Salvatore Rubino, Università degli Studi di Sassari, Dipartimento di Scienze Biomediche, Sassari, Italy, who performed conjugation experiments for this study.
